# Informing Equity-Oriented Approaches to Postpartum Care Following Severe Maternal Morbidity Events: A Qualitative Descriptive Study Protocol

**DOI:** 10.1177/23333936261448865

**Published:** 2026-05-13

**Authors:** Susan M. Jack, Daniel J. Atkinson, Amanda Hicks, Rosie Deol, Rohan D’Souza, Benicio N. Frey, Sheryl Green, Luis Francisco Ramos-Lima, Laura N. Anderson, Elizabeth K. Darling, Amie Davis, Danielle Rice, Meredith Vanstone, Giulia M. Muraca

**Affiliations:** 1 McMaster University, Hamilton, ON, Canada; 2 St. Joseph’s Healthcare Hamilton, ON, Canada

**Keywords:** severe maternal morbidity, women’s health, qualitative description, Canada

## Abstract

Severe maternal morbidity (SMM) encompasses life-threatening complications during pregnancy, childbirth, or the postpartum period and is associated with profound physical, psychological, and social consequences. Individuals affected by SMM often report fragmented postpartum care and unmet emotional needs, underscoring the need for equity-oriented approaches. Embedded within the SERENE (Supportive, Evidence-informed, Responsive, and Equity-oriented prevention and care model to reduce the incidence and NEgative impacts of severe maternal morbidity) research program, this protocol describes a qualitative descriptive study (2024–2027) that aims to generate data grounded in the experiences of people affected by SMM and health care providers to inform the development of postpartum care pathways, practice guidance and educational interventions. Using purposeful sampling, we will conduct semi-structured interviews with multiple participant groups including individuals who have experienced SMM, involved family members, and health care professionals across Ontario, Canada. Findings will inform the co-development of practice guidance, a care pathway and an educational intervention to enhance provider confidence in delivering equity-oriented care. Ethical considerations include use of a participant safety protocol to navigate discussion of sensitive topics and trauma-informed supports for research staff. This study will generate actionable insights to strengthen postpartum care systems and reduce health inequities for individuals recovering from SMM.

## Background

The term severe maternal morbidity (SMM) captures a heterogeneous set of conditions associated with severe illness, debilitation, prolonged hospitalization, long-term disability, and high case fatality among pregnant, birthing and postpartum individuals (Dzakpasu et al., 2020; Kilpatrick et al., 2014). In high-income countries where maternal mortality is low, SMM is recognized as a more appropriate marker of quality of maternity care than maternal mortality (Geller et al., 2018; Mengistu et al., 2020). SMM can result in negative short- and long-term health and socioeconomic impacts for individuals who give birth as well as their children and families (Dzakpasu et al., 2019). Short-term impacts may include increased risk of maternal mortality, infant death, adverse perinatal outcomes like stillbirth, preterm birth, or low birth weight, and higher health care costs given the expense of life-saving treatments and interventions (Dzakpasu et al., 2019; Phibbs et al., 2022). There can also be profound long-term negative impacts of SMM on maternal mental health, parent-infant bonding, child development, family functioning, chronic comorbidity, and economic sufficiency (Dzakpasu et al., 2019; Knight et al., 2016; Phibbs et al., 2022).

Globally, SMM, which is sometimes operationalized using the maternal near-miss framework, affects an estimated 1.4% of live births, with higher rates observed in low-and middle-income countries (Firoz et al., 2022). Data indicates that the incidence of SMM in Canada is increasing (17.5 per 1,000 births in 2022 compared with 13.8 per 1,000 births in 2007; Joseph et al., 2010; Public Health Agency of Canada, 2024) and is high relative to the rate of SMM in other high-income countries (11.4 per 1,000 births; Henderson et al., 2025). Importantly, the incidence of SMM across high-income countries varies widely based on several factors including maternal age, race, ethnicity, Indigenous status and identity, low income status, gender identity, sexual orientation, disability and exposure to intimate partner violence (Ayala Quintanilla et al., 2023; Brown et al., 2021; Dzakpasu et al., 2020; Hailu et al., 2022; Obedin-Maliver & Makadon, 2016; Owais et al., 2020; Phillips-Beck et al., 2019; Snelgrove et al., 2021).

Suboptimal maternal health care, including delayed or missed diagnosis, misdiagnosis, or delayed treatment, is known to exacerbate the severity of SMM outcomes (Geller et al., 2018). Furthermore, an individual’s experience of SMM may be influenced by factors other than those related specifically to the SMM event, including system-level issues, which can be seen reflected in an individual’s perceived sense of safety in conjunction with care provision or availability and accessibility of high-quality health care. Consequently, many individuals view SMM experiences as being fearsome, disempowering, painful, and/or life-threatening events with negative effects being further amplified by inadequate care (Furuta et al., 2014; Hicks et al., n.d.; Wang et al., 2021). Given the intensity and enduring psychological impact reported by many individuals following SMM events, it is important to consider these experiences within the broader context of trauma and structural violence.

The definition of trauma is nuanced (Dalenberg et al., 2017), but the Substance Abuse and Mental Health Services Administration (2014) offers a widely used framework that conceptualizes trauma through the three “E’s”: the event, the individual’s experience of the event, and its effects. Accordingly, trauma reflects the interplay between a potentially harmful or threatening event, the individual’s interpretation of that event, and its enduring physical, psychological or social impacts. Within the context of childbirth, some individuals describe their birthing experience itself as traumatic (Beck, 2004; Reed et al., 2017); however, SMM introduces layers of threat, loss of control, and vulnerability that may profoundly shape how the event is experienced and the health consequences that follow. For individuals who have maternal near-death experiences, they are at increased risk for suicidal thoughts and depression (Beck, 2004; Small et al., 2020; von Rosen et al., 2021).

Certain aspects of maternal care may exacerbate the trauma associated with SMM, particularly gaps in continuity of care and poor provider communication, including patients feeling unheard, which undermine trust (Hansen et al., 2024; Wang et al., 2021). Many individuals report dissatisfaction with care, citing delays in assessment, diagnosis, or treatment; inappropriate management; limited information; poor therapeutic relationships; lack of qualified providers; and under-resourced systems (Norhayati et al., 2015). Perceived discrimination or clinician bias toward those experiencing social and economic disadvantage, Black individuals, and those who use substances further contributes to distrust (Hansen et al., 2024; Hoang et al., 2023). These patterns can be understood of as manifestations of structural violence (Farmer et al., 2006), wherein social, economic, and health system arrangements systematically constrain access to timely, respectful, and high-quality care, often disproportionately affecting equity-deserving populations seeking maternal health care services (Crear-Perry et al., 2021)

Continuity of care, particularly following hospital discharge, is critical, yet some individuals report feeling abandoned and lacking access to needed resources (Abdollahpour et al., 2019; Knight et al., 2016; Latt et al., 2023). Although people who experience SMM typically require ongoing monitoring, treatment, and follow-up (Knight et al., 2016; Niles et al., 2024), stigma and trauma-related responses can lead to avoidance of care, as health care settings may trigger distress, activate memories of the traumatic event, leading to flashbacks, anxiety and fear (Furuta et al., 2014). This combination of disrupted continuity and avoidance further complicates postpartum recovery, particularly when emotional and psychological needs remain unmet.

People who have experienced SMM report that health care providers often prioritize physical recovery over emotional and psychological well-being, to the detriment of mental health (Furuta et al., 2014; Knight et al., 2016; Latt et al., 2023; Niles et al., 2024). The emotional consequences and psychological trauma of SMM can persist for years and may overshadow positive experiences of new parenthood, leaving some individuals hesitant to consider subsequent pregnancies (Wang et al., 2021). These long-term effects can impair individuals’ ability to parent, maintain employment, and engage fully in social and personal life (Furuta et al., 2014). At the health system level, delayed or avoided care is associated with more severe complications, more complex and costly interventions (Phibbs et al., 2022), and increased strain on emergency and acute care services (Wang et al., 2021), while also perpetuating health inequities (Furuta et al., 2014; Hoang et al., 2023; Kolahdooz et al., 2016; Wang et al., 2021). These impacts underscore the need for equity-oriented health care in the immediate postpartum period for people who have experienced SMM (Knight et al., 2016; Niles et al., 2024). Equity-oriented care integrates culturally safe care, trauma- and violence-informed care, and harm reduction at both interpersonal and organizational levels (Varcoe et al., 2019).

This paper introduces the SERENE (Supportive, Evidence-informed, Responsive, and Equity-oriented prevention and care model to reduce the incidence and NEgative impacts of severe maternal morbidity) program of research and the research foci of its four embedded project Pillars. This is followed by the study protocol for a qualitative descriptive study (Pillar 3) that will be conducted to deepen our understanding of the multi-disciplinary and equity-oriented approaches to service delivery and education that are needed to support health care providers in safely identifying and responding to individuals’ needs following an SMM event.

## The SERENE Program of Research

SERENE is a collaborative and multidisciplinary research program (2024–2027) in Ontario, Canada that aims to generate knowledge to: (a) inform the prevention of SMM; (b) strengthen care for those who experience SMM; and (c) inform the development of equity-oriented practices and education supports for individuals and families affected by SMM as well as the professionals who provide their care. Independent but interrelated studies will be conducted across four research program Pillars. Pillar 1 will involve secondary data analysis of population-based datasets in Ontario to better understand the types of SMM and their implications, as well as identify the populations at greater increased risk for these events. Pillar 2 will include the development of the Canadian Obstetric Survey System in Ontario (CanOSS Ontario; Malhamé et al., 2025) and the conduct of a province-wide program aimed at reducing SMM. Pillar 3, which is the primary focus of this protocol paper, will involve deepening understandings of individuals’ experiences and expectations of health care following an SMM event. Pillar 4 will provide mental health expert advice to Pillars 1–3 and will focus on developing strategies to both improve awareness of the links between SMM and perinatal health. In a commitment to safeguarding the emotional and psychological safety of researchers and trainees (Jack et al., 2023) across Pillars, clinician-researchers in Pillar 4 will also provide reflective supervision and trauma-oriented training workshops as required.

### SERENE’s Lived Experience Advisory Network

Partnership with diverse individuals who have lived and/or living experience of a health issue is rapidly, and appropriately, becoming the norm in Canadian health research processes (Birnie et al., 2025; Michalak et al., 2023). This shift supports an equitable valuation of experiential knowledge alongside other ways of knowing and understanding (Roche et al., 2020) with such cooperation being justified from a democratic-ethical perspective both in terms of people having influence over decisions that affect their lives (Thompson et al., 2014), and from capacity-building perspectives, with patients being engaged in the healthcare system as co-producers of knowledge (Manafo et al., 2018). By engaging with people who have lived experience of illness in processes of health research, a “triple aim” impact becomes possible, with improvements in (1) patients’ experiences of care, (2) patients’ outcomes both at individual and population levels, and (3) per capita healthcare expenditures (Manafo et al., 2018).

Patient engagement is not monolithic as a practice in research, and there are various stages in which people may be engaged in research processes, and different levels of participation that help define the fullness of patient engagement and which modulate the power dynamics that often accompany patient partner-researcher relationships (Adams et al., 2024; Birnie et al., 2025). Adams et al. (2024) provide a framework that outlines how patient-partners may be engaged in the research planning, conduct, interpretation or dissemination stages. They further define that levels of engagement may vary and be defined by the direction of communication between patient partners and researchers, as well as by the degree of decision-making responsibility that patient partners have. Moreover, there is increasing recognition of the necessity for trauma-informed, intersectional, and critically-reflexive approaches to patient partnership, with these approaches highlighting how multiple social identities interact with processes of oppression and discrimination, and being considerate of how health care systems and services may (usually inadvertently) activate trauma among those they seek to serve (Birnie et al., 2025).

Establishing and supporting SERENE’s patient engagement effort–known as its “Lived Experience Advisory Network” (or “LEAN”) – has been a priority from the beginning of the SERENE research program. As such, SERENE’s Project Manager (DJA) also serves as the Chair of the Patient Engagement Committee and was tasked with locating and assembling a diverse group of approximately 12 people who have lived and living experience of SMM to work with the SERENE team at all stages of the SERENE project. While communication with the LEAN and Pillar-related LEAN involvement is year-round, the LEAN itself gathers on a quarterly basis to discuss all elements of the SERENE project. LEAN members join the group, attend meetings and, if need be, depart the LEAN in accordance with their personal capacities to participate, although most LEAN members have maintained their membership for more than a year. Striking a balance between maximizing the plurality of LEAN membership (benefiting the diversity of people who can weigh in on the above) and ensuring that all LEAN members have ample opportunities to speak, converse with each other, and be heard in safety has inclined us toward our target of having 12 standing members. To promote the safety of members, LEAN meetings and communication approaches are facilitated and supported by a specialist in trauma- and violence-informed approaches.

To date, SERENE’s LEAN has been involved with planning, conduct and interpretation stages across all four of SERENE’s research pillars by ensuring that (a) the research questions we are asking are useful and appropriate; (b) the research approaches we use are productive and unlikely to cause harm to participants; and that (c) our interpretations of preliminary findings are realistic and meaningful. Specifically, as consultants for Pillar 3, LEAN members have partnered with the qualitative research team in designing approaches to participant recruitment, developing data generation tools and protocols, and ensuring that the participant safety protocol is suitable for use during interviews in cases of participant distress. Some of the members were engaged in the synthesis of study findings included in a qualitative systematic review to describe the health care experiences and expectations of individuals with SMM, enhancing the meaningfulness and credibility of those interpretations. Moving forward, collaboration with the LEAN will be ongoing and it is anticipated that members will be deeply involved with SERENE’s development of educational interventions and will guide knowledge dissemination efforts as novel understandings come into focus, such that these understandings can be communicated effectively with the community at large.

### Pillar 3 Qualitative Study Objectives and Research Questions

Given the extent and degree of health and social challenges experienced by individuals and their family members during the recovery process following an SMM event, there is a critical need to determine how postpartum healthcare systems can incorporate the provision of equity-oriented care to survivors to minimize re-traumatization and to maximize their recovery and health promotion efforts. Qualitative research provides a powerful means to explore complex and context-dependent health issues by illuminating the experiences, needs, and recommendations of individuals who have endured SMM. Conceptually, these insights can deepen understandings of how individuals affected by SMM experience care, thus generating new ways of thinking about service delivery and therapeutic relationships. Instrumentally, qualitative findings can directly inform the design of care pathways, practice guidance and educational interventions that are responsive to women’s articulated needs and priorities (Jack, 2006). The two primary objectives of this descriptive qualitative study are to formatively develop: (1) a model of postpartum care to identify and respond to the needs of individuals who have experienced SMM; and (2) an education intervention curriculum to train pre-service and in-service health care professionals about the needs and care expectations of this population. These objectives will be accomplished in two study phases. In Phase 1, a qualitative descriptive study will be conducted to document affected individuals’ and health care professionals’ experiences and recommendations for the receipt and delivery of postpartum care following an SMM event. In Phase 2, data from Phase 1 will be used to generate recommendations for care pathways, develop and disseminate practice guidance resources for physicians, midwives and nurses working in both acute and community-based settings, and develop and test for acceptability an educational intervention to support health care professionals working with this population to provide equity-oriented care.

The EPPiC (Emphasis-Purposeful sample-Phenomenon of interest-Context) framework (Jack & Phoenix, 2022; Moisey et al., 2022) for generating questions for applied qualitative health research studies was used to craft the study’s overarching research questions:

Among individuals who have experienced an SMM event and health care professionals who provide or manage services for this population within Ontario, Canada:

1. What are their perceptions of, and experiences with, the supports and services that are currently available for individuals and their family members who have experienced an SMM event?
2. What are the types and characteristics of services and supports needed in the postpartum period?
3. What knowledge and skills do health care professionals need to safely and confidently identify and respond to individuals who have experienced an SMM event?

## Explanation and Justification of Methods

### Research Design

The principles of qualitative description (Bradshaw et al., 2017) will inform all purposeful sampling, data generation and analytic decisions. Within applied qualitative health research studies, this study design provides a framework that allows for the identification and description of participants’ perceptions and experiences of the phenomenon under study and articulation of their recommendations for change and future recommendations. The level of exploration in this type of descriptive qualitative research typically focuses on “discovering the who, what, where and why of events or experiences” (Bradshaw et al., 2017, p. 3). Findings from this type of methodologically pragmatic and applied research are often used in the formative development of novel interventions or programs (Bradshaw et al., 2017). Additionally, a “problem-practice-needs” framework (van Meijel et al., 2004) will be used as sensitizing concepts in the data generation and analysis phases of the study to generate qualitative data that can be utilized to develop practice-oriented recommendations for care and education that specifically address the needs and priorities of individuals who experienced an SMM event, and to provide insights on what concepts health care providers may need additional education about.

Consistent with qualitative description, this study is underpinned by a relativist ontology, which assumes that multiple, subjective realities exist and are shaped by individuals’ experiences and interpretations of the phenomenon under study (Bradshaw et al., 2017). Epistemologically, our study is also informed by subjectivism, recognizing that knowledge is co-constructed through the interaction between the participants and researchers (during the interviews) rather than discovered as an objective truth (Bradshaw et al., 2017). As such, participants’ accounts will be understood as both descriptive and interpretive representations of their experiences of receiving or providing care related to SMM events. This positioning will acknowledge our active role as researchers in interpreting the data while privileging participants’ perspectives through the use of rich, verbatim accounts to support analytic interpretations.

### Context

Within applied qualitative health research, the human or social phenomenon being studied will be influenced by the social, geographic, or temporal context in which it is embedded (Jack & Phoenix, 2022). Therefore, to facilitate the future transferability of the findings, it is critical for researchers to provide a detailed description of the context (Stalmeijer et al., 2024). For this study, experiences of SMM postpartum care are being explored within the context of Ontario’s universal and publicly funded healthcare system. The key features of prenatal, labor and delivery, and postpartum care in Ontario are summarized in Table 1.

**Table 1.** Context of Prenatal, Labor and Delivery, and Postpartum Care in Ontario.

Health care system component	Characteristics
Universal access to health care	• Essential health and medical services in Ontario are publicly funded through the Ontario Health Insurance Plan (OHIP) – including prenatal care, care during labor and delivery, and postpartum care and supports. • Private options (such as a private hospital room) may be available at additional cost.
Maternity care providers	• Obstetricians manage all aspects of pregnancy, labor and delivery, and postpartum care and are designated providers for higher-risk pregnancies, typically in hospital settings. • Family physicians provide early and routine prenatal care and may perform deliveries for low-risk pregnancies. They routinely provide longitudinal comprehensive care in the postpartum period and beyond. • Registered midwives offer comprehensive care for low-risk pregnancies, and provide continuity of care during pregnancy, delivery (hospital settings or home-births) and in the first 6 weeks postpartum. • Nurse practitioners may provide prenatal or postpartum care as part of primary care practices or in organized team-based programs.
Prenatal care	• Primary care providers, midwives or obstetricians typically offer prenatal care (including routine physical examinations, diagnostic testing as required, and screening for maternal and fetal health issues). • Community-based agencies (including public health) generally offer prenatal classes, health education, and support services for nutrition, mental health, and breastfeeding/infant feeding.
Labor and delivery care	• Women and pregnant individuals have access to multiple birthing options: home-births (for low-risk pregnancies), hospital births, or birthing centers.
Postpartum care	• Deliveries in hospital typically include a 24–48 hr stay in hospital following an uncomplicated vaginal delivery (with slightly longer stays for a cesarean section birth, depending on health status of birthing parent and infant). • A follow-up check is generally arranged approximately 6 weeks after delivery with primary care provider or obstetrician. Family physicians will often see families in the postpartum period associated with newborn care and may provide ongoing care across multiple visits in the 6-week postpartum period. For midwifery-managed care, midwives typically provide ongoing care and support for up to 6 weeks postpartum. • Information on all births (along with associated risks) are recorded via a universal postpartum screening program and communicated to one of Ontario’s 29 public health units. • Following an additional assessment of risks, families may be eligible for a range of postpartum care supports through public health, including breastfeeding support or home visits.
Public health and specialized services	• Ontario’s 29 public health units are mandated to deliver the Healthy Babies Healthy Children program which includes universal postpartum screening to identify families at-risk and to provide home visits from public health nurses and family home visitors to families with identified risk (with children age 0 to school entry). • Public health units also provide prenatal education, online postpartum information, breastfeeding supports and parenting programs. • The Nurse-Family Partnership program (10 public health units) is a nurse home visitation program for young (<24 years) pregnant and parenting individuals experiencing social and economic disadvantage. Home visits start early in pregnancy and continue until the child’s second birthday.
Specialized services	• Specialized services to address mental health needs of birthing parents during pregnancy and in the postpartum period (including assessment and treatment for postpartum mood and anxiety disorders) are typically coordinated through tertiary care centers. • Private options (such as a private psychotherapy) may be available at additional cost.

### Sampling

Purposeful sampling, or the inclusion of individuals who can provide rich and detailed accounts of receiving care following an SMM event or delivering care will be invited to participate. This purposeful sample will consist of five distinct data sources: (1) individuals who have experienced an SMM event (*n* = 40); (2) the most involved family member or informal support (when possible) of the individual with SMM (*n* = 20); (3) health care professionals who provide care or services to individuals with SMM (*n* = 24); (4) first-line staff (e.g., referral clerks, receptionists; *n* = 5); and (5) senior managers or administrators responsible for the development and implementation of postpartum coordinated care pathways or services in the Hamilton region (*n* = 5). A phased approach to participant recruitment and data generation will be employed. The inclusion criteria for each data source group are summarized in Table 2. Our *a priori* sample size estimate is 94 individuals. Sample size determination was driven by the concept of information power (Malterud et al., 2016). Information power refers to the idea that the more relevant, specific, and rich the information that participants can contribute to answering the study’s aim, the fewer participants are needed; conversely, lower information power requires a larger sample to achieve sufficient analytic depth. Given this, we judged that a total sample of 94 purposively distributed among women with SMM, their partners, health-care providers and administrators will yield sufficient information power because the study has a focused, clinically relevant aim, high participant specificity, and will generate rich data through in-depth interviews analyzed by an experienced qualitative team. Conversely, the deliberate inclusion of multiple distinct stakeholder groups and the need to triangulate diverse perspectives may introduce some heterogeneity that requires a larger overall sample to capture meaningful variation and achieve analytic sufficiency across groups, which justifies the planned total of 94.

**Table 2.** Inclusion Criteria by Data Source.

Data source	Inclusion criteria
Phase 1	
Individuals who have experienced an SMM event	• People who self-report of having experienced an SMM event*; • in the period of January 2015 to December 2025; • who received care for childbirth, miscarriage or pregnancy termination in Ontario; • who are 16 years of age or older; • who are at least 6 months postpartum; • who express a readiness to discuss their experiences of SMM and the health care they received; and • who can complete an in-depth interview in English.
Most involved family member or informal support	• People who are identified as a “most-involved” partner, family member or friend by an individual who experienced an SMM event*; • who attended or observed at least one health care encounter related to the diagnosis, treatment or response to the SMM event; and • who can complete an in-depth interview in English.
Health care professionals	• People who are a regulated health care provider in Ontario** • who have provided postpartum health care services in Ontario; • who have provided postpartum health care services during or since January 2020; and • who have provided health care services to at least one individual who experienced an SMM event* during the perinatal period or the first year postpartum.
Phase 2	
First-line staff	• People who are employed as referral clerks or receptionists in family practice or primary care settings in Ontario.
Senior managers or administrators	• People who are employed as senior managers and/or administrators; and • who are responsible for the development and implementation of coordinated care pathways or services within an across local communities in Ontario.

* SMM events include: severe preeclampsia, eclampsia, or HELLP syndrome; severe hemorrhage; maternal ICU admission; surgical complications (including complications of obstetric surgery and procedures, evacuation of incisional hematoma with red blood cell transfusion, reclosure of cesarean wound with red blood cell transfusion, or repair of bladder, urethra or intestine); unplanned hysterectomy; sepsis; embolism, shock, or disseminated intravascular coagulation; assisted ventilation; cardiac conditions (including cardiac complications of anesthesia, cardiomyopathy, cardiac arrest and resuscitation, myocardial infarction, or pulmonary edema and heart failure); acute renal failure; severe uterine rupture; cerebrovascular events; acute abdomen; acute fatty liver with red blood cell or plasma transfusion; adult respiratory distress syndrome; cerebral edema or coma; pulmonary, cardiac, and central nervous system complications of anesthesia during pregnancy, puerperium, or labor and delivery; sickle cell crisis; status asthmaticus; status epilepticus; or surgical or manual correction of inverted uterus for vaginal births. **Regulated health care providers include: Medical Doctor, Registered Nurse, Registered Nurse (Extended Class), Registered Midwife, Occupational Therapist, Physiotherapist, Registered Psychotherapist, and Registered Psychologist.

With respect to the individuals who have experienced SMM, efforts will be made to identify and recruit a diverse range of people, including participants from equity-deserving groups (e.g., individuals with a disability, individuals from the 2SLGBTQ+ community). Maximum variation sampling will be used to purposefully introduce diversity into the sample based on the variables of birth location (e.g., delivery in hospital, home birth, birthing center, social identity, or type of SMM event) and timing of SMM event (e.g., while in hospital, onset within 42 days postpartum).

### Recruitment

Given the rarity of SMM events in the population, multiple recruitment strategies will be used to identify individuals who have experienced an SMM event as well as those health care professionals who provide postpartum care. Using snowball sampling (Palinkas et al., 2015), the first strategy will involve SERENE research team members generating a list and extending an invitation to participate in the study to Ontario health care professionals who provide prenatal, obstetrical or postpartum health care services and are likely to have provided care to an individual with SMM. Patient-oriented study recruitment materials will also be distributed to these health care professionals with a request for them to share them with current or past patients who may meet study eligibility criteria. Health care professionals will also be invited to post a study recruitment poster within their clinic waiting or treatment rooms. These study recruitment posters will also be posted within community-based organizations (e.g., EarlyON Centres, libraries, recreation centers) that are frequently accessed by caregivers and parents with young children. All print and electronic resources will include a QR code that will link to a REDCap electronic data capture tool (Harris et al., 2009; Harris et al., 2019) hosted at McMaster University to confirm client eligibility and a direct contact to a member of the research team. Finally, members of SERENE’s LEAN and program leads of closed networks including the Public Health Nursing Practice, Research and Education Program (www.phnprep.ca), the 29 Ontario Healthy Babies Healthy Children and 10 Nurse-Family Partnership programs, the Greater Hamilton Health Network Primary Care Network and community-based metal health networks will be provided with a digital version of the recruitment materials to circulate to staff within their organizations or to relevant patient populations.

All individuals who experienced an SMM event who consent to participate in the study will also be asked to identify if they had a most involved family member (e.g., partner, sibling, parent, friend) who supported them during the SMM event or assisted them to access health care during the first 42 days postpartum. If the study participant expresses their consent for their family member or friend to learn more about the study, the research coordinator will either: (a) ask them to obtain permission from the family member/friend to directly share their contact information with the study team or (b) provide the study recruitment poster with the participant to share, with guidance for family member/friend to directly contact the study team.

While these strategies to recruit via closed networks will be implemented, the Pillar 3 research team explicitly decided to avoid public advertising of the study on common social media platforms. Within qualitative health research there is substantive evidence documenting the increasing frequency and escalating risks of enrolling inauthentic (also labeled as fraudulent or impostor) participants (Ridge et al., 2023; Sharma et al., 2024). In a scoping review of 23 studies to map strategies in health research to detect and counteract fraudulent responses, most studies employed some form of online or web-based recruitment strategies (Comachio et al., 2025). As many studies provide study participants an honorarium, it has been suggested that individuals who purposefully misrepresent their eligibility criteria do so for financial gain (Husted et al., 2025). By not advertising details of compensation in certain online contexts, we hope to mitigate the risk of enrolling inauthentic participants. However, the risk is not fully mitigated given that paper and electronic versions of the study recruitment posters will be circulated via the methods described above across multiple organizations, and individuals viewing these forms may share study information with individuals who wish to misrepresent their eligibility to participate. Therefore, despite the risk of inadvertently excluding some participants (Drysdale et al., 2023), the research team will employ additional risk mitigation strategies (Bandiera et al., 2025; Husted et al., 2025) at the outset of interviews. If there are multiple suspicious occurrences, the interview may be postponed for team review at the discretion of the interviewer. In cases when a potential participant is deemed likely to be inauthentic, the circumstances of that individuals’ exclusion from the study will be documented in the study’s research log.

### Data Generation

Data generation procedures for Phase 1 have been fully developed. In contrast, the data generation and analytic procedures for Phase 2 will be designed after Phase 1 is completed, as they will be directly informed by the findings and outputs generated and developed in Phase 1. This phased approach ensures that Phase 2 is methodologically responsive and grounded in the empirical insights generated earlier in this study.

To describe their experiences, identify their priority needs, and share recommendations for practice and education, all study participants will be invited to participate in a single in-depth, semi-structured interview lasting 60 to 90 min. Although partners or spouses may both be enrolled in the study, dyadic interviews will not be conducted due to ethical concerns regarding potential power imbalances and participant safety. In intimate relationships, unequal dynamics related to gender, age, or dependency may inhibit open discussion or compromise one partner’s comfort in disclosing sensitive experiences (Szulc & King, 2022). Individual interviews are therefore a more appropriate method to ensure participant autonomy, safety, and the opportunity for honest and uninfluenced reflection. Interviews will be scheduled times that are mutually convenient for the participant and the researcher, and participants will be offered the option to complete the interview via telephone or videoconference (for individuals living within the Hamilton region, the option for an in-person interview will be offered). Permission to audio record each interview will be sought. Given the number of interviews to complete, six research team members (inclusive of the principal investigator, three research coordinators, and two doctoral trainees) completed an orientation on the study interview process and objectives. These interviewers all have previous experience conducting qualitative interviews and have SMM content expertise; additionally, four interviewers (RD, PG, AH, SMJ) have extensive nursing experience working with populations of pregnant or parenting women through their respective work in public health, labor and delivery, or the neonatal intensive care unit.

Distinct semi-structured interview guides (see Supplemental File A) were developed to guide the discussions with each of the three data sources (i.e., individuals with experiences of SMM, their family/friend who supported them during their recovery, and health care professionals) participating in Phase 1. To facilitate data source triangulation and the use of the constant comparative technique during analysis, common concepts were explored across all interviews (Table 3). Implementation science is a useful approach to understanding critical gaps between what should happen and what actually happens in healthcare contexts (Hamm et al., 2021), and we have employed elements of the Consolidated Framework for Implementation Research (CFIR; Damschroder et al., 2022) to structure several lines of inquiry in our interview guides. The CFIR’s Outer and Inner Settings are both helpful concepts here, with the Outer Setting exploring factors that are external to the organizations that provide care (such as issues of access, available resources, sociocultural determinants of health, and care coordination), and the Inner Setting pertaining to structures, cultures and resources within healthcare facilities and the teams who provide maternal care (such as values, characteristics and qualities of healthcare programs and facilities, and relationships between patients and health care providers). Furthermore, a summary of evidence on women’s expectations of care from health care providers (Furuta et al., 2014; Hicks et al., 2025; Norhayati et al., 2015) has been incorporated into the interview guide for health care providers, along with associated questions that explore health care providers’ perspectives on this summary and its implications regarding the delivery of compassionate, holistic, individualized, trauma-informed care. Being located mid-interview, it is our hope that this summary does not unduly influence participants’ responses to prior questions while also creating a space where these understandings may be discussed and used as jumping-off points for challenges health care providers face in providing the kinds of care that women and birthing individuals have indicated that they want and need. Preliminary drafts of interview guides were shared with LEAN members to ensure that questions and probes are appropriate lines of inquiry, and that content, tone and language are unlikely to be retraumatizing for participants who have experienced severe maternal morbidity, and LEAN members’ feedback and recommendations were used to finalize the interview guide questions. All study participants will also complete a demographic survey.

**Table 3.** Central Interview Concepts by Data Source.

Central concept	Data sources		
	Individuals with SMM	Family member/friend	Health care professional
• Experience and expectations of care proximal to the SMM event	X	X	
• Services and supports in the SMM recovery period	X	X	
• Reimagining a postpartum care system to meet the needs of individuals and families	X	X	X
• Individual characteristics	X	X	X
• Program characteristics	X	X	X
• Outer setting characteristics	X	X	X
• Inner setting characteristics	X	X	X
• Support for families	X	X	
• Education of health care professionals	X	X	X
• Final insights or recommendations	X	X	X

At the end of each interview, a field note form will be completed to capture information on study logistics and contextual information about personal or environmental factors that may have influenced engagement of either the researcher or the participant during the interview. The field note form also includes space to document if the emotional safety pathway was utilized (and outcomes), new or important ideas arising during the interview, reflexive insights and general operational notes.

### Data Analysis

Demographic data will be summarized using descriptive statistics. Audio recordings of each interview will be transcribed verbatim with identifying information removed. A process of team-based, in-person rapid qualitative analysis will be used to summarize and synthesize the interview data across the three distinct data sets (Campbell et al., 2024; Hamilton & Finley, 2019). Rapid qualitative analysis is a structured yet efficient approach that uses summary templates, data matrices, and iterative team discussions to summarize and synthesize qualitative data while maintaining analytic rigor. Developed for use within implementation science, this process of rapid qualitative analysis facilitates a research team’s capacity to identify problems, needs, and recommendations that can directly inform decision-making and intervention design (Hamilton & Finley, 2019). This analytic approach is particularly appropriate for qualitative descriptive studies which aim to provide comprehensive summaries of experiences and practical insights rather than more in-depth theoretical interpretations (Bradshaw et al., 2017). Consistent with Bradshaw et al. (2017) guidance for analysis within qualitative descriptive studies, this approach allows for the integration of multiple perspectives, enhances data credibility and dependability through real-time analytic consensus, and supports the production of actionable findings.

To conduct the analysis of the dataset, a team of up to 12 analysts (including research staff, study investigators, PhD trainees and 2-3 LEAN members) will convene for a 3 day in-person analysis meeting. Prior to the meeting, three templates (one for each data source) with a list of domains developed from the interview questions will be developed and pilot tested by two members of the research team to test for consistency in data extraction. At the analysis event, transcripts will be evenly distributed across team members. Each analyst will begin by reading their transcripts in their entirety. Then each analyst will extract key quotes and summarize transcript data by topic domain within the template. All summarized data for each domain will then be organized together, with an analyst using content and frequency analytic techniques to develop a synthesis of findings for each domain.

### Data Integration to Inform Phase 2 Activities

Findings from Phase 1 of this qualitative study and from a qualitative systematic review of women’s experiences and expectations of care following an SMM event (Hicks et al., 2025) will be integrated and used to identify content, processes and structures to include in: (1) a postpartum care pathway for health care professionals and organizations; (2) practice guidance resources and (3) an educational intervention to increase health care professionals’ skill and confidence in providing equity-oriented health care to this population of pregnant and parenting individuals. Feedback on draft versions of the care pathway will be sought from Phase 2 study participants (e.g., front-line staff, program administrators/managers), SERENE research team investigators, trainees and LEAN members.

### Ethical Considerations

All study procedures and data generation tools have been approved by the Hamilton Integrated Research Ethics Board (REB #18164). Informed consent will be obtained prior to participation in any interview, with consent being reviewed together either in-person, over the telephone, or over Zoom. Participants will be again reminded of their rights (including the right to withdraw from the study at any time, for any reason) immediately prior to the conduct of any interview or focus group.

**Figure 1.**
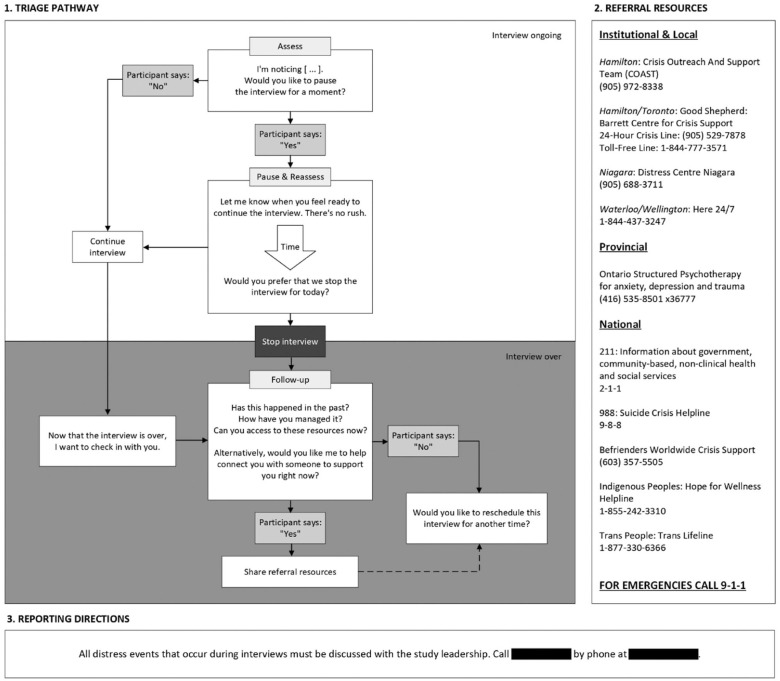
Participant safety protocol.

Ensuring participant safety and well-being during interviews is of high importance, particularly as potentially sensitive and traumatic experiences will be discussed (Jack et al., 2023). Research team interviewers have been trained in recognizing signs of participant distress and versed in trauma- and violence informed care principles and will employ a Participant Safety Protocol (see Figure 1) during all interviews. The Participant Safety Protocol suggests a triage pathway for managing unexpected or adverse emotional events, clearly lists referral pathways to appropriate support services, and encourages research team members to discuss all distress events with study leadership.

The emotional safety of research team members will also be a priority in the conduct of this research, given the sensitivity of the topic and the risk of secondary distress to interviewers (Jack et al., 2023). This will be supported through research team participation in a SERENE Interviewer Community of Practice, which will meet regularly, per the needs of members of the research team. This support structure, which will be co-managed by the study’s research coordinator and a clinician-scientist representative from Pillar 4, is designed to be an ongoing space for general support where interviewers can discuss challenges, acknowledge the emotional toll that can result from exposure to discussions of traumatic subjects, and strategize ways that this qualitative data collection work can be conducted safely and sustainably. Furthermore, in cases of acute interviewer distress, a “phone-out tree” structure has been formalized, whereby research team members can contact (a) the research coordinator, (b) the study’s principal investigator, and/or (c) a clinician-scientist representative of Pillar 4 to discuss emotional challenges and to seek support at any time that it is needed.

### Approaches to Supporting Qualitative Rigor

Application of the four criteria in Lincoln and Guba’s (1985) trustworthiness framework is a methodologically congruent approach to promoting rigor during the conduct of a qualitative descriptive study (Bradshaw et al., 2017). Strategies to enhance the credibility, confirmability, dependability and transferability of findings from this proposed study are summarized in Table 4.

**Table 4.** Strategies to Support Qualitative Rigor.

Criteria	Strategies
Credibility	• Triangulation of three data sources • Researcher triangulation by including interviewers and analysts with diverse professional backgrounds and perspectives • Training to advance interviewer skills to establish rapport and demonstrate genuine compassion during interviews as strategies to facilitate trust development and information sharing • Research team engages in personal, interpersonal, and methodologic reflexivity practices
Confirmability	• A research log (audit trail) used to record all sampling, data generation and data analytic processes and decisions • Field notes recorded following each interview • Reports and future articles reporting findings will include direct quotations from participants
Dependability	• Interviewer reviewing transcript against audio file for accuracy • Team-based approach to rapid qualitative analysis that includes opportunities for discussion and iterative development of categories of findings • Pilot testing of data summary template
Transferability	• Detailed descriptions of context and participants to be included in reports and publications • Purposeful sampling, including approaches to maximum variation sampling

#### Reflexivity

Reflexivity is an important process for enhancing the credibility and trustworthiness of qualitative research findings. Through a set of ongoing, collaborative, and multifaceted practices, qualitative researchers can acknowledge, explain, and even leverage their contextually derived subjectivity (biases and assumptions), and account for how such factors influence their inquiry (Olmos-Vega et al., 2023). For the proposed study, the core group of researchers (DJA, RD, AH, SMJ) involved in conceptualization, methodologic decision-making, data generation, data analysis, and interpretation bring important and diverse perspectives shaped by their different social identities, including gender, race, age, education, geographic location in Ontario, discipline, and experience with pregnancy/birth. They are committed to implementing three types of reflexivity throughout the study: personal, interpersonal, and methodologic. Personal reflexivity requires each researcher to disclose their background and position(s) in relation to the research and to continuously examine their expectations, assumptions, and reactions to research contexts, participants, and data through reflective processes (Olmos-Vega et al., 2023). In this case, each researcher will create a social identity map (Jacobson & Mustafa, 2019) where they identify their various social positions, reflect on how these positions impact their perspectives, and critically consider how their social identity could influence their interactions with the study participants and throughout the data generation and analysis processes. Reflexive journalling (Karcher et al., 2024) is integrated with the social identity mapping activity to promote ongoing and emerging personal reflections. Interpersonal reflexivity requires a more nuanced appreciation for relational power dynamics in the research context (Olmos-Vega et al., 2023). Field notes and memos completed by interviewers following each interview are used to capture critical interpersonal dynamics and insights (Phillippi & Lauderdale, 2017). Team discussions will occur regularly to reflect on and, if needed, respond to these dynamics within their relationships with each other; between researcher and participant; and within the socio-political context of the study. Utilizing trauma-informed research approaches promotes safety necessary for these discussions among the team (Isobel, 2021) and documenting discussions using a reflexivity log will promote transparency. Lastly, methodologic reflexivity asks researchers to critically examine the impact of their methodologic decisions, questioning whether they are ethical, rigorous, and aligned with their paradigmatic orientation (Olmos-Vega et al., 2023). This is again an ongoing practice, and the credibility of findings is strengthened in qualitative research when methodologic decisions are made through collaboration among researchers with diverse backgrounds and expertise (Olmos-Vega et al., 2023). Importantly, the research team for this study have already established a culture that promotes trust and curiosity during bi-weekly meetings with protected time for collaborative decision-making and team-based reflection.

## Dissemination Plan

We will employ a multi-modal, audience-specific dissemination strategy to ensure the findings of this qualitative study are accessible, useful, and actionable for diverse knowledge users. Consistent with conventional academic dissemination, we will prepare a series of peer-reviewed publications and present findings at relevant national and international conferences in nursing, midwifery, obstetrics, public health and maternal-child health.

Recognizing that academic channels alone have limited impact on practice and education change, we will also develop a suite of applied knowledge-translation products. These will include short research briefs summarizing key findings in accessible language and tailored for health care professionals, policymakers, and other community partners. In partnership with the Public Health Nursing Practice, Research and Education Program (PHN-PREP; www.phnprep.ca), we will co-develop practice guidance resources that integrate the study’s findings into practical recommendations for public health nurses and other postpartum care providers supporting individuals who have experienced SMM. These resources will be disseminated through PHN-PREP networks and featured in their monthly webinar series (approximate reach 400–500 professionals/session).

We will further use the study findings as foundational components for an educational intervention aimed at enhancing health care providers’ knowledge, skills, and confidence in delivering equity-oriented postpartum care to individuals who have experienced SMM. Finally, we will collaborate with regional postpartum care leaders to co-develop and refine postpartum care pathways, informed by the study results, to support their integration into local practice and system planning. This comprehensive dissemination approach is designed to improve knowledge translation efforts beyond academic publication, ensuring meaningful impacts on education, practice and service delivery for individuals recovering from SMM.

## Discussion

SMM is a critical public health concern in Canada. It is understood that the incidence of SMM events in Canada is increasing, and that the occurrence of SMM often results in profound short- and long-term socioeconomic and health impacts for those who experience it – including psychological trauma, chronic comorbidity, and life-long debilitation. Furthermore, impacts of SMM are known to extend beyond individuals who experience SMM events in hindering maternal-infant bonding, family functioning, and the economic sufficiency of affected families. Recognizing that SMM-related trauma is too-often amplified by suboptimal care, there is an urgent need for better understandings of the health care experiences and expectations of people who experience SMM events, and for the promotion of compassionate, accessible, and truly helpful approaches to supporting individuals who experience SMM in the aftermath of these devastating events. The methods outlined in this protocol are well aligned with the study aims of developing equity-oriented care pathways and educational interventions to enhance postpartum care systems and to assist providers in better meeting the needs of individuals who have experienced SMM, and the needs of these individuals’ family members. The use of a qualitative descriptive design ensures that the study will capture rich yet contextually grounded accounts of experiences, expectations, and recommendations for improvement from multiple perspectives, including individuals affected by SMM, their family members, and health care professionals who care for those who experience SMM. Purposeful sampling will enable the inclusion of diverse voices and experiences, including individuals from equity-deserving groups – which is critical for informing care models that address systemic inequities.

Furthermore, interviews across these diverse sources will generate actionable insights into gaps in current care and education, as well as opportunities for improvement, while the integration of trauma-and violence-informed approaches throughout this research project safeguards the well-being of both participants and members of the research team. The use of novel team-based approaches to rapid qualitative analysis will produce timely, rigorous results that can inform Phase 2 activities. Finally, by working in partnership with SERENE’s LEAN while also embedding multiple reflexivity practices and strategies to promote trustworthiness, the study will yield findings that are methodologically sound and practically relevant. Ultimately, these methods collectively position the research to meet its objectives of advancing equity-oriented postpartum care and supporting health care professionals to deliver compassionate, psychologically safe, and responsive services to all people who are recovering from SMM events.

## Supplemental Material

sj-docx-1-gqn-10.1177_23333936261448865 – Supplemental material for Informing Equity-Oriented Approaches to Postpartum Care Following Severe Maternal Morbidity Events: A Qualitative Descriptive Study Protocol Supplemental material, sj-docx-1-gqn-10.1177_23333936261448865 for Informing Equity-Oriented Approaches to Postpartum Care Following Severe Maternal Morbidity Events: A Qualitative Descriptive Study Protocol by Susan M. Jack, Daniel J. Atkinson, Amanda Hicks, Rosie Deol, Rohan D’Souza, Benicio N. Frey, Sheryl Green, Luis Francisco Ramos-Lima, Laura N. Anderson, Elizabeth K. Darling, Amie Davis, Danielle Rice, Meredith Vanstone and Giulia M. Muraca in Global Qualitative Nursing Research
