# Supplementary material for: Informing Equity-Oriented Approaches to Postpartum Care Following Severe Maternal Morbidity Events: A Qualitative Descriptive Study Protocol
Source: Glob Qual Nurs Res. 2026 May 13;13:23333936261448865. doi: 10.1177/23333936261448865 (PMC13173341; doi:10.1177/23333936261448865)
Supplement: sj-docx-1-gqn-10.1177_23333936261448865 – Supplemental material for Informing Equity-Oriented Approaches to Postpartum Care Following Severe Maternal Morbidity Events: A Qualitative Descriptive Study Protocol [file sj-docx-1-gqn-10.1177_23333936261448865.docx]

**Supplementary File A – Interview Guides**

**SERENE** (Supportive, Evidence-informed, Responsive, and Equity-oriented prevention and care model to reduce the incidence and NEgative impacts of severe maternal morbidity)

**Pillar 3:** **Informing Equity-Oriented and Trauma- and Violence-Informed Approaches to Care Following an SMM Event**

**Semi-Structured Interview Guide**

**People who have experienced an SMM event: Individual Interview**

**Interview Logistics**

| **Participant ID #** |  |
| --- | --- |
| **Interview Date**  (month/day/year) |  |
| **Interviewer** |  |
| **Interview start time** |  |
| **Interview end time** |  |
| **Interview number**  (e.g., 1 of 1, 1 of 2, 2 of 2) |  |
| **Interview mode**  (e.g., Zoom, In-person) |  |
| **Demographic survey completed** |  |
| **Type of $50 gift card requested** |  |
| **Additional Notes** |  |

**Introduction**

Hello, my name is (*name*) and I am the (*position*) on this study.

We wanted to talk to you because we understand you experienced what we call severe maternal morbidity, a long name for serious health challenges that can happen while you are pregnant or after you give birth. Some examples of this are severe blood loss, life-threatening heart conditions, surgical complications, or maternal intensive care unit admission. First, I want to check in about your eligibility - does this sound like something you experienced?

Ok, thank you. So, in our study we are trying to understand the experiences of health care that people had after they experienced things like *(what the participant told you they experienced, or give an example or two)*. For example, what you wish would have gone better, and what was really helpful to you during your recovery. This could include experiences you had in the hospital or with your family doctor, or interactions you had with your midwife or other health care support workers. There are no right or wrong answers to these questions, we’re just interested in learning from you about what you experienced and what you think could have gone better. Does that make sense?

We are hoping that by the end of this study we will be able to provide recommendations to health care providers about what services are needed, and who should provide them, and how they could be offered in a way that would really help people experiencing these types of pregnancy issues. So any time throughout our conversation that you have a thought about how things could have gone better, please tell me what your recommendations are, so we can communicate them to the people who design and run these programs.

Ok, now I’ll tell you a bit about what we are going to do today. We will talk for about an hour, maybe less or maybe more. I will do my best to keep us on track and generally on time, but if you have to go at any time, or take a break, that’s no problem. I’m going to record our conversation so I can make sure I capture all of your ideas. If we lose our connection at any time, don’t worry – I will wait for you to re-connect and we will resume our interview. If you want to skip any questions, you can. If you want to end the interview, you can do that at any time too. Does that sound ok? Do you have any questions before I begin?

**Interview Questions**

**For prioritizing your interview time, please note questions’ highlighting:**

**High priority questions** (always ask, probe as appropriate)

**Medium priority questions** (ask if you feel inquiry is warranted, e.g.,

if thrust of question has not been addressed earlier in the interview)

**Low priority questions** (do not ask unless there is a very specific reason)

1. **Confirming participant details**
2. I’m going to start by confirming a few details about you and your experience. Could you please tell me:
   1. What year you were born in?
   2. What year your SMM happened?
   3. And what province you had your health care in after your SMM experience?
3. **Experience and expectations of care proximal to the SMM**
4. Thinking back, could you briefly share with me a description of the severe complications you experienced during pregnancy, at the time of delivery, or in the period after pregnancy?
   1. Sensitively prompt for outcome of the pregnancy.
5. Please tell me about the care you received when you were experiencing *(severe complications described above).*
6. What could have been done differently that would have made your experience of care during this pregnancy, delivery or in the period after you gave birth, better?
   1. Prompt if necessary: more comfortable, or safer?

1. **Services and supports in the SMM recovery period**
2. In the first 6 weeks after you gave birth, what types of physical or mental health concerns happened that were related to the SMM you experienced?
   1. During the first 12-months after you gave birth, what types of physical or mental health concerns did you experience?
3. In the 12 months following the birth of your infant, what professional medical or social supports did you use to get help to address your concerns (e.g., appointments with family physician, midwife, or public health services)?
   1. Do you have a regular family doctor or nurse practitioner?
   2. Were you able to be in contact with this person while experiencing [specific health needs]?
   3. How did they respond to you?
4. In the 12 months following the birth of your infant, what supports were most helpful in meeting your needs?
   1. Why? What made these supports helpful?
   2. Did (supports mentioned in Question 2) meet your needs?
5. In the 12 months following the birth of your infant, what supports were not helpful?
   1. Why?
   2. What supports were needed, but were not available? Or what kind of care and support did you need most, yet did not receive?
6. During this period, when you were focusing on your recovery, what was your experience of navigating the health care system to get the care and services you needed?
   1. What factors made it easier for you to get the care or services you needed?
   2. What factors made it difficult for you to get the care or services you needed?
7. **Re-imagining a postpartum care system to meet the needs of individuals and families following serious complications in pregnancy or the early postpartum period**

Following serious complications during pregnancy, childbirth or after birth, many individuals need to seek a range of health care services to address ongoing physical and mental health needs, as well as supports to navigate other challenges. An important goal of this study is to understand what types of support individuals need during this period, and how, where, and by whom these services could be offered.

***Characteristics of individuals***

1. What types of services do you think are essential to meet the needs of women and birthing individuals who experience severe complications during pregnancy, childbirth or after giving birth?

***Program Characteristics***

1. I’d like to ask you some more specific questions to understand your health needs in the postpartum period:
   1. Where should these services be offered? (e.g., a specialized clinic located in a physical space such as a hospital, through all family physicians, through home visits, or a hybrid approach)?
   2. How would you prefer to access these services? (e.g., virtual visits, in-person clinic visits, in-person home visits)
   3. What are important qualities that you look for in the health care that you receive (e.g., consistency, thoroughness, compassion)?
   4. What qualities do you not like in your health care?
   5. What types of support or care would you expect to receive from your health care provider (e.g., mental health support, physical health support, health promotion, support for parenting, occupational health)?
   6. How do you feel about the idea of a trained person from your community (e.g., someone from your cultural group, religious group, or local neighbourhood) providing some degree of emotional, mental or physical support to you at home? Would you prefer that to going to a hospital in some cases? Why or why not?
   7. In an ideal situation, how long after pregnancy do you think services should continue to be offered? (e.g., physical rehabilitation, wound care, consults for bleeding or pain, lactation and parenting support)

***Outer Setting***

1. What factors make it difficult to access and use postpartum services?
2. What factors would make postpartum services and supports more accessible for individuals who experience these types of severe complications?
3. How could postpartum care services help to address potential barriers to accessing and utilizing the supports they offer?

***Inner Setting***

1. What are the most important values or characteristics you would want a postpartum care program to uphold?
2. How would you describe an ideal relationship between you and members of the postpartum health care team?
   1. Probe for elements such as level of trust, communication, continuity of care.
3. How would you like your pregnancy care provider (e.g., midwife, Maternal-Fetal Medicine specialist, or obstetrician) to address the specific challenges you faced recovering from severe pregnancy-related complications?
   1. How would you like your primary care provider (e.g., family doctor, or nurse practitioner) to address these challenges?
   2. How would you like pregnancy-related specialists (e.g. lactation consultants, or perinatal mental health specialists) to address these challenges?
4. What qualities of the health care setting – particularly when accessing primary care or community-based services – would make you feel safer, more comfortable and better supported when accessing care in the postpartum period?
5. **Support for families**
6. Do you think your partner/spouse, or a close family member who was involved in your pregnancy could have benefited from any support?
   1. What kind of support?
7. Do you think any other family members such as children could have benefitted from any support?
   1. What kind of support?
8. **Education of Health Care Professionals**

One of our goals will be to develop a short course for health care professionals, including doctors, nurses and midwives, as well as for students in those fields, to learn about severe maternal morbidity and the health care experiences and needs of women and birthing people who experienced severe complications in pregnancy.

1. What are the main messages from your experience that you would want all health care professionals to understand?
2. What recommendations would you give to them on how they can provide high-quality, compassionate and safe care to women/individuals following these types of severe complications?
3. **Final thoughts**

Before we finish today, do you have any additional recommendations on how the health care system can safely and compassionately meet the postpartum needs of women and birthing people who experienced severe complications in pregnancy, childbirth or the postpartum period?

Is there anything I didn’t ask you that you think is important for us to know?

Lastly, as part of this study, we’re also interested in exploring the perspectives of partners, family members, and friends who have supported women and birthing people who have experienced an SMM. May I ask if anyone in particular supported you in the aftermath of your SMM, and if so, if you would be comfortable with them sharing their own perspectives on the health care you received as you recovered? If there was someone specific who supported you, and if this sounds alright to you, I would be glad to provide you with information about this study to share with them so that they can contact us if they’re interested in participating.

Thank you so much for talking to me today and sharing your experiences. Your insights and recommendations will be very helpful in developing better services to meet the needs of women, birthing people, and their families. If we have follow-up questions as we work on this project, would it be ok for us to contact you again? If so, would you prefer a call, text, or email?

**SERENE** (Supportive, Evidence-informed, Responsive, and Equity-oriented prevention and care model to reduce the incidence and NEgative impacts of severe maternal morbidity)

**Pillar 3:** **Informing Equity-Oriented and Trauma- and Violence-Informed Approaches to Care Following an SMM Event**

**Semi-Structured Interview Guide**

**Most involved family member of a person who experienced SMM: Individual Interview**

**Interview Logistics**

| **Participant ID #** |  |
| --- | --- |
| **Interview Date**  (month/day/year) |  |
| **Interviewer** |  |
| **Interview start time** |  |
| **Interview end time** |  |
| **Demographic survey completed** |  |
| **Type of $50 gift card requested** |  |
| **Additional Notes** |  |

**Introduction**

Hello, my name is (*name*) and I am the (*position*) on this study.

In our study, we are trying to understand the experiences of health care that people had after they experienced serious health challenges while they were pregnant or after they gave birth. We wanted to talk to you about your experiences of providing support to your (*wife/partner/child/friend/family member*) who experienced severe complications during their pregnancy, childbirth or in the period shortly after pregnancy.

We are hoping that by the end of this study we will be able to provide recommendations to health care providers about what services are needed, and who should provide them, and how they could be offered in a way that would really help people experiencing these types of pregnancy issues. So any time throughout our conversation that you have a thought about how things could have gone better, please tell me what your recommendations are, so we can communicate them to the people who design and run these programs.

Ok, now I’ll tell you a bit about what we are going to do today. We will talk for about an hour, maybe less or maybe more. I will do my best to keep us on track and generally on time, but if you have to go at any time, or take a break, that’s no problem. I’m going to record our conversation so I can make sure I capture all of your ideas. If we lose our connection at any time, don’t worry – I will wait for you to re-connect and we will resume our interview. If you want to skip any questions, you can. If you want to end the interview, you can do that at any time too. Does that sound ok? Do you have any questions before I begin?

**Interview Questions**

**For prioritizing your interview time, please note questions’ highlighting:**

**High priority questions** (always ask, probe as appropriate)

**Medium priority questions** (ask if you feel inquiry is warranted, e.g.,

if thrust of question has not been addressed earlier in the interview)

**Low priority questions** (do not ask unless there is a very specific reason)

1. **Experience and expectations of care proximal to the SMM**
2. Thinking back, could you briefly share with me your experience of supporting your (*wife/partner/child/friend/family member*) when they experienced severe complications during pregnancy, childbirth or in the postpartum period?
   1. Sensitively prompt for outcome of the pregnancy.
3. What did you think about the quality of care they received during this period of time when these serious complications were being treated?
4. As their support person, can you describe to me how health care professionals interacted with you and responded to your presence?
5. **Services and supports in the SMM recovery period**
6. In the postpartum period, what type of support did you provide to your (*wife/partner/child/friend/family member*)?
7. What professional medical or social supports did you accompany your (*wife/partner/child/friend/family member*) to?
8. Which supports were most helpful in meeting their needs?
   1. Why? What made these supports helpful?
9. What supports were not helpful? What supports were not available?
   1. Why?
10. During this period, what was your experience of navigating the health care system to get the care and services your (*wife/partner/child/friend/family member*) needed?
    1. What factors made it easier for get the care or services they needed?
    2. What factors made it difficult to get the care or services they needed?
11. **Re-imagining a postpartum care system to meet the needs of individuals and families following serious complications in pregnancy or the postpartum period.**

Following serious complications during pregnancy, childbirth or the first six weeks postpartum, many individuals need to seek a range of health care services to address ongoing physical and mental health needs in the postpartum period, as well as supports to navigate other challenges. An important goal of this study is to understand what types of support individuals need during this period and how, where, and by whom these services could be offered.

***Characteristics of individuals***

1. What types of services are essential to meet the needs of women and birthing people who experienced severe complications during pregnancy, childbirth or after giving birth?
2. What types of services/supports should be available for their (*spouses/partners/family members/friends*)?
3. Do you think any other family members such as children could have benefitted from any support?
   1. What kind of support?

***Program Characteristics***

1. To identify, assess, and address the diverse health needs of women and birthing people who experienced complications during pregnancy, childbirth or the postpartum period:

a. Where should these services be offered (e.g., a specialized clinic located in a physical space such as a hospital, through all family physicians, through home visits, or a hybrid approach)?

b. What options for how these services could be delivered would appeal to you? (e.g. virtual visits, in-person clinic visits, in-person home visits)

c. What are important qualities you look for in the health care needed by women and birthing people who experienced severe complications (e.g. consistency, thoroughness, compassion)?

***Outer Setting***

1. What factors make it difficult to access and use postpartum services?
2. What factors would make postpartum services and supports more accessible for individuals who experienced these types of severe complications?

***Inner Setting***

1. What are the most important values or characteristics that you would want a postpartum care program to uphold?
2. How would you describe an ideal relationship between you and members of the postpartum health care team?
   1. Probe for elements such as level of trust, communication, continuity of care.
3. What qualities of the health care setting would make your (*wife/partner/child/ friend/family member*) feel safer, more comfortable and better supported when accessing care in the postpartum period?
4. **Education of Health Care Professionals**

One of our goals will be to develop a short course for health care professionals, including doctors, nurses and midwives, as well as for students in those fields, to learn about severe maternal morbidity and the health care experiences and needs of women and birthing people who experienced severe complications in pregnancy.

1. What are three main take-away messages from your experience that you would want all health care professionals to understand?
2. What recommendations would you give to them on how they can provide high-quality, compassionate and safe care to women and birthing people following these types of severe complications?
3. **Final thoughts**

Before we finish today, do you have any additional recommendations on how the healthcare system can safely and compassionately meet the postpartum needs of women and birthing people who experienced severe complications in pregnancy, childbirth or the postpartum period? Additionally, do you have any recommendations for their partners, family members, or friends?

Is there anything I didn’t ask you that you think is important for us to know?

Thank you so much for talking to me today and sharing your experiences. Your insights and recommendations will be very helpful in developing better services to meet the needs of women, birthing people, and their families.

**SERENE** (Supportive, Evidence-informed, Responsive, and Equity-oriented prevention and care model to reduce the incidence and NEgative impacts of severe maternal morbidity)

**Pillar 3:** **Informing Equity-Oriented and Trauma- and Violence-Informed Approaches to Care Following an SMM Event**

**Interview Guide**

**Health Care Professionals: Individual Interview**

**Interview Logistics**

| **Participant ID #** |  |
| --- | --- |
| **Interview Date**  (month/day/year) |  |
| **Interviewer** |  |
| **Interview start time** |  |
| **Interview end time** |  |
| **Interview number**  (e.g., 1 of 1, 1 of 2, 2 of 2) |  |
| **Interview mode**  (e.g., Zoom, In-person) |  |
| **Demographic survey completed** |  |
| **Type of $50 gift card requested** |  |
| **Additional Notes** |  |

**Introduction**

Hello, my name is (*name*) and I am the (*position*) on this study.

We wanted to talk to you because we are interested in your experiences providing care to individuals who have experienced severe maternal morbidity events – severe maternal morbidity, or SMM, events being unexpected outcomes related to pregnancy, labour, childbirth and the postpartum period resulting in severe illness, prolonged hospitalization and long-term disability that occur during pregnancy and up to 42 days postpartum. Through this research, we are hoping to understand the unique health and social care needs of this population, and to explore how their postpartum needs could be best and most feasibly met through the publicly funded health care system. Your experiences and perspectives about this topic are very important, so any time throughout our conversation that you have thoughts about how things could be done better, please tell me what your recommendations are. There are no right or wrong answers to these questions, and you can decline to answer any question that makes you uncomfortable. Does that sound alright to you?

Ok, so today we will talk for about an hour, and I will do my best to keep us on track and generally on time, but if you have to go at any time, or take a break, that’s no problem. I’m going to audio record our conversation so I can make sure I capture all your ideas. If we lose our connection at any time, don’t worry – I will wait for you to re-connect and we will resume our interview. If you want to skip any questions, you can. If you want to end the interview, you can do that at any time too. Does that sound ok? Do you have any questions before I begin?

**Interview Questions**

1. **Re-imagining a postpartum care system to meet the needs of individuals and families following serious complications in pregnancy or the postpartum period**

Following serious complications during pregnancy, childbirth or the first six weeks postpartum, many individuals need to seek a range of health care services. They do this to address ongoing physical and mental health needs in the postpartum period, as well as supports to navigate other challenges. An important goal of this study is to understand what types of support individuals need during these periods and how, where, and by whom these services could be offered.

***Characteristics of individuals***

1. What kinds of SMM do you see in your professional practice?
2. What factors facilitate your ability to recognize, assess and respond to the health care needs in the postpartum period of women and birthing people who have experienced SMM? (Probe for knowledge, skills, confidence, etc.)
3. What are some of the biggest challenges you have experienced in providing postpartum care to these individuals?
   1. How do these challenges impact your approach to care delivery with this population?

***Program Characteristics***

1. The next few questions relate to identifying, assessing, and addressing the diverse health needs of women and birthing people who experienced SMM or severe complications during pregnancy, childbirth or the postpartum period:

a. What specific types of support or interventions are essential in providing postpartum care to this population? What postpartum needs or expectations of this population do you perceive are currently not being met?

b. What features of a postpartum care program would you consider to be critical to making it feasible for health care professionals or systems to deliver, and for patients and birthing people to access?

c. Where should these services be offered from (e.g., a specialized clinic located in a physical space such as a hospital, through all family physicians, through home visits, or a hybrid approach)?

d. What options for how these services could be delivered would appeal to you? (e.g., virtual visits, in-person visits)

***Outer Setting***

1. What are the most significant external factors (e.g., community resources, access to speciality care) that influence your ability to provide adequate postpartum care to women and birthing people with SMM?
2. How do the social determinants of health (e.g., socioeconomic status, geographical location, access to family support) influence the postpartum care of women with SMM?
3. How would you describe the collaboration among healthcare providers and healthcare organizations in coordinating care for this population?
   1. What realistic changes could be made to improve coordination between care providers and care systems?

***Inner Setting***

1. Within your health care setting, what systems or supports exist to address the complex postpartum needs of women and birthing people with a history of SMM?
2. What barriers do you experience within your organization when you’re trying to provide comprehensive care to this population of individuals/patients?
3. I am going to share my screen now [**content at end of interview guide**] and you will see a slide with a summary of findings from qualitative studies of women and birthing people who have experienced SMM and their expectations regarding their interactions with health care professionals. Please let me know when you have finished reading the slide’s contents.
   1. How does this summary resonate with your understandings of the care needs of women and birthing people who are recovering from SMM experiences?
   2. In your view, how well does the current system align with these expectations, and where do you see the most significant gaps?
   3. What individual factors do you think affect healthcare professionals’ ability to deliver the compassionate and trauma-informed care that these women and birthing people are seeking?

(If asked, ‘**trauma-informed care**’ is *an approach that recognizes the widespread impact of trauma and aims to create a safe and supportive environment for individuals who have experienced it. This care model focuses on understanding trauma's effects, avoiding re-traumatization, and promoting healing and recovery through compassionate and patient-centered practices.*)

- 1. From an organizational perspective, what factors do you perceive limit the delivery of holistic care that includes both physical and mental health support for women and birthing people with SMM?
  2. What could be done at an organizational or system level to better support integrated mental and physical health support in the postpartum care offered to these women and birthing people?
  3. How do organizational or systemic factors either support or hinder culturally-sensitive and individualized care for women and birthing people who have experienced SMM?
  4. Based on your experience, what changes would be needed at both the individual and organizational levels to deliver care that more closely aligns with women’s and birthing people’s expectations in the postpartum period?
  5. What supports, resources or policy changes do you think are necessary to address the identified barriers and provide trauma-informed and equity-oriented care for women and birthing people with SMM?

(If asked, ‘**equity-oriented care**’ is *an approach that aims to reduce the negative health effects of: multiple, intersecting forms of racism, discrimination and stigma; structural inequities and structural violence; and the frequent mismatches between usual approaches to care and the needs of people most impacted by health and social inequities.*)

1. **Education of Health Care Professionals**

One of our goals will be to develop a short course for health care professionals, including doctors, nurses and midwives, as well as for students in those fields, to learn about severe maternal morbidity and the health care experiences and needs of women and birthing people who experienced severe complications in pregnancy.

1. What knowledge is essential for healthcare providers to be able to identify, assess and respond to the needs of women and birthing people who have experienced SMM?
2. What educational strategies could be used to increase health care professionals’ skills and confidence in delivering care that is underpinned by the principles of trauma- and violence-informed care?

(If asked, the **principles of trauma- and violence-informed care** include: *understanding trauma and violence's effects on people’s lives and behaviour; creating emotionally-, culturally-, and physically-safe environments for all clients; fostering opportunities for choice, collaboration and connection for and with clients; and using strengths-based and capacity-building approaches to support clients.*)

1. **Final thoughts**

Before we finish today, do you have any additional recommendations on how the health care system can safely and compassionately meet the postpartum needs of women and birthing people who experienced severe complications in pregnancy, childbirth or the postpartum period?

Is there anything I didn’t ask you that you think is important for us to know?

If we have any follow-up questions in the future, would it be alright if we contacted you again?

Lastly, once the educational modules have been developed based on the recommendations that you and other health care professionals have suggested, would you be interested in helping to pilot-test these educational materials?

Thank you so much for talking to me today and sharing your experiences. Your insights and recommendations will be very helpful in developing better services to meet the needs of women, other birthing individuals and their families.

***Shared screen content from Question A.10.***

**Summary of Evidence on Women’s Expectations of Care from Health Care Providers**

**1. Compassionate and Empathetic Communication**

Women often expect healthcare professionals to acknowledge and validate the traumatic nature of their experience. Studies indicate that women desire empathy and compassion in their interactions, and want clinicians to acknowledge the psychological impact of SMM and its life-altering nature. The importance of non-judgmental, attentive listening is frequently cited. Women value providers who listen actively and express understanding, which helps build trust and makes them feel safe to discuss their fears and their physical or emotional needs.

**2. Clear and Transparent Information**

Many women report feeling overwhelmed by the complexity of SMM, and they expect clear, jargon-free explanations from their healthcare providers. They want comprehensive information about their condition, its causes, and potential long-term effects. Transparency about treatment options, possible complications, and recovery timelines is particularly valued, as women often feel fear and uncertainty about their future health and potential for future pregnancies.

**3. Follow-up and Continuity of Care**

Women with SMM express a need for more structured and consistent follow-up care beyond the immediate postpartum period. They often feel that their care ends prematurely and that they are left to navigate complex health issues alone once discharged. Many expect a coordinated approach, where their primary care provider, obstetrician, and any specialists communicate effectively with each other to ensure seamless and continuous care. This is especially critical for women who experience ongoing complications.

**4. Holistic Care That Includes Mental Health Support**

Emotional and mental health support is consistently highlighted as a critical need after experiencing SMM. Women report that while physical recovery is often prioritized, the emotional toll of SMM can be equally profound, and they expect mental health screenings and support to be part of their postpartum care. Access to mental health professionals, or at least referrals to appropriate services, is a common expectation. Women want providers to proactively ask about mental health and recognize that trauma recovery is part of postpartum care.

**5. Patient Empowerment and Involvement in Care Decisions**

Women with SMM often express a desire to be actively involved in decision-making processes regarding their care. This includes understanding the rationale for specific treatments and having a say in their care plan. Empowerment through shared decision-making helps them regain a sense of control after the traumatic experience of SMM, where they may have felt vulnerable or helpless.

**6. Respect for Individual Experiences and Cultural Sensitivity**

Women want healthcare providers to respect the individuality of their experiences and to avoid treating SMM patients as a homogeneous group. They expect care that is sensitive to their unique cultural, personal, and socioeconomic contexts. Tailored care that aligns with their values and beliefs can improve comfort levels and help them feel respected, especially if their SMM experience has resulted in significant lifestyle or identity changes.
